# Rett syndrome: interferon-γ to the rescue?

**DOI:** 10.1038/s44321-024-00154-7

**Published:** 2024-11-04

**Authors:** Richard R Meehan, Sari Pennings

**Affiliations:** 1 MRC Human Genetics Unit, Institute of Genetics and Cancer, University of Edinburgh, Edinburgh, EH4 2XU UK; 2 Centre for Cardiovascular Science, Institute for Neuroscience and Cardiovascular Research, University of Edinburgh, Edinburgh, EH16 4TJ UK

**Keywords:** Genetics, Gene Therapy & Genetic Disease, Neuroscience

## Abstract

R. Meehan and S. Pennings discuss the recent work from Frasca et al, in this issue of *EMBO Mol Med*, that shows that neural precursor cells rescue symptoms of Rett syndrome by activation of the Interferon γ pathway.

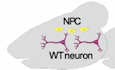

## Teaser

Rett syndrome is a severe neurodevelopmental disorder in girls, underpinned by mutations in the X-linked gene *MECP2*. In their recent work (Frasca et al, 2024), Frasca and colleagues identified a novel pathway involving interferon-gamma (IFNγ) that could pave the way to potential therapies.

The initial identification of the methyl-CpG binding protein, MeCP2, resulted from biochemical assays designed to detect DNA methylation-dependent DNA binding activities in mammalian nuclear extracts (Lewis et al, 1992; Meehan et al, 1992a). This was spurred by early in vitro and ectopic transcriptional analyses supporting the idea that transcriptional repression by promoter DNA methylation may be mediated by methyl-CpG binding factors (Meehan et al, 1992b). More recent observations suggest that the number of genes fitting this profile in vivo is relatively small, as around 1% of CpG island promoters are methylated in somatic tissues (Smith et al, 2012). These promoters mostly correspond to genes normally expressed in the germline which function to repress retrotransposon activities, and are not exclusively repressed by MeCP2 (Crichton et al, 2014). So, what is the role of MeCP2?

MeCP2 belongs to the family of methyl-CpG binding domain (MBD) proteins, with partially redundant binding specificities and expression patterns (Tillotson and Bird, 2020). MeCP2 binding to methylated CpGs leads to the recruitment of transcription repressive complexes that include histone deacetylases, which remove histone acetylation necessary for gene activation. *MECP2*’s importance first came to light when it was shown to map to the X-chromosome and underlie Rett syndrome, characterized by a progressive inability from 6 to 18 months of age to coordinate eye and body movements along with speech impairments, autism, and reduced lifespan in females (Amir et al, 1999; Tillotson and Bird, 2020). Many *MECP2* mutations have since been identified, which cause male fetal mortality but allow apparently normal tissue development in randomly X-inactivated heterozygous (Het) females. Mouse models were subsequently established, in which *Mecp2* deletion in male mice phenocopied many aspects of human Rett syndrome, while mutations in the *Mecp2* methyl-CpG binding domain and transcription repression domains resulted in disease phenotypes (Tillotson and Bird, 2020). A bridge model proposes that MeCP2 recruits the NCoR1/2 repressor complex to methylated DNA, acting as a global repressor of gene expression in the brain (Tillotson and Bird, 2020).

In mouse models of Rett syndrome, the expression of normal levels of wild-type MeCP2 or specifically truncated forms of MeCP2, through genetic activation or virus-mediated delivery to the brains of mutant mice has been shown to reverse symptoms and restore many normal functions (Tillotson et al, 2017; Tillotson and Bird, 2020). This incredibly important observation has been a stimulus for research into pharmaceutical, epigenome editing, and cell biology therapies that could potentially improve the outcomes and quality of life for Rett syndrome patients (Qian et al, 2023; Tillotson and Bird, 2020). However, due to the wide spectrum of *MECP2* mutations and attributed functions, it has proven difficult to pinpoint precisely when and which biological pathways are affected.

**Figure 1:**
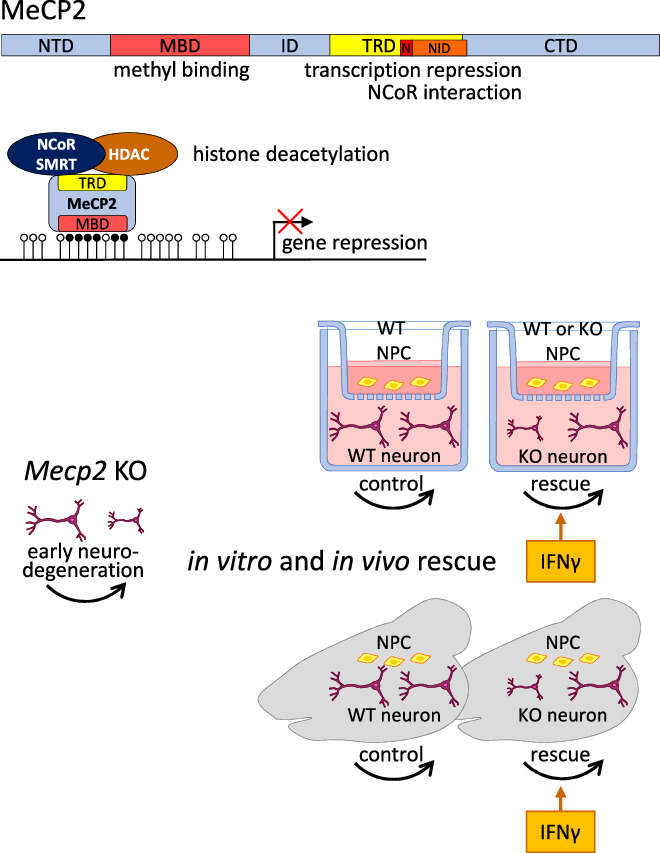
MeCP2 structure, function and proposed mutant rescue. Top: MeCP2 protein (486 aa) domain structure consisting of NTD N-terminal domain, MBD methyl binding domain, ID intervening domain, TRD transcription repression domain, NID NCoR interaction domain, CTD C-terminal domain, N nuclear localization signal. Middle: diagram of MeCP2 bound to methylated CpGs (black lollipops), recruiting transcription repressive complexes nuclear receptor corepressor 1 (NCoR)/silencing mediator for retinoid and thyroid hormone receptors (SMRT) and histone deacetylases (HDAC) to a gene neighborhood. Bottom: Mecp2 knockout (KO) neurons are defective but based on Frasca et al (2024) can be rescued by co-culture with neural progenitor cells (NPC, wild-type (WT) or KO) in vitro (trans-well co-culture experiments) or when NPC are transplanted into the brain (grey) of Mecp2 KO mice. Interferon γ (IFNγ) was shown to exert regenerative effects on Mecp2 KO neurons, attributed to an IFN pathway response to damage signals. WT neurons controls showed no effect.

In a potentially significant advance, Frasca and colleagues target a neuronal loss-of-function end-point in *Mecp2* mutant mice via brain transplantation of neural progenitor cells (NPCs). This rescues Rett syndrome symptoms partly through activation of the interferon-gamma (IFNγ) pathway in NPC transplanted *Mecp2* mutant mice. Deficits in Rett neurons are similarly rescued in co-culturing experiments (Fig. 1).

The investigators first employed in vitro co-cultures with NPCs from 6- to 8-week-old mice to chart their impact on *Mecp2* knockout (KO) neurons, characterized by reduced dendritic complexity and synaptic puncta density compared to wild-type (WT) neurons. This phenotype was rescued by NPCs, but not by fibroblast-derived NIH3T3 cells. Interestingly, WT and *Mecp2* KO NPCs were equally effective in increasing pre- and post-synaptic densities in KO neurons, which may have critical implications for human cell-based therapeutic treatments. In addition, conditioned media from NPCs co-cultured with KO neurons exclusively rescued defects in Rett neurons. This could reveal NPCs’ capacity to sense an altered neuron environment and release beneficial factors that restore deficits in Rett neurons.

Impressively, the authors further demonstrated that NPC transplantation ameliorated Rett-like symptoms in *Mecp2* deficient mice. Around 10 million NPCs were transplanted by intra-cisterna magna injection in symptomatic 45/47-day-old *Mecp2* KO mice. The lifespan of *Mecp2* KO mice increased, and several motor and cognitive functions were rescued. About 2% of the transplanted cells persisted in the KO brain at the end of the assessment and exhibited an immature phenotype. One interpretation is that the transplanted NPCs are beneficial through the release of soluble factors in a transient paracrine mode, rather than through cellular replacement of differentiated tissues. NPC grafting at a later stage, in 180-day-old *Mecp2* Het mice, recovered the memory defect but did not improve the mice's general well-being and motor skills, which correlated with the more limited grafting efficiency and transplanted cell survival observed in this case.

*MECP2* loss of function is reported to induce subtle but widespread gene deregulation in neural tissues, which may result from its role as a global transcriptional regulator. Transcriptional profiling and bioinformatics analysis of NPC transplanted *Mecp2* KO cerebella and controls suggested the deregulation of several pathways associated with immune response, including the pathway related to interferon-gamma (IFNγ). Similar observations were made in KO hippocampi after transplantation.

Encouraged by these results, IFNγ’s ability to rescue altered dendritic complexity and synaptic puncta density in Rett neurons was tested. The highest dose of IFNγ (100 ng/ml) proved effective in restoring deficits in *Mecp2* KO and Het neurons, while no detrimental effect was observed on WT neurons. In line with these results, a single intracerebral injection of IFNγ in KO and Het animals rescued short-term motor and memory impairments, yet respiratory defects were not improved.

These results raise many intriguing questions and possible applications. Does the study mimic brain development conditions, where NPC could exhibit a protective effect that would decrease once NPCs become more restricted? IFN-γ is known to have antiviral, immunoregulatory, and anti-tumor properties through receptor-mediated pathways in its target cells, including microglia (Kann et al, 2022). These resident macrophages impact brain development, homeostasis and critically respond to injury and repair. Of note, a strong interaction between social behavior and IFN-γ-driven responses has been suggested in rodents (Filiano et al, 2016). How does IFNγ improve the functionality of Rett neurons, and could therapies be designed to improve outcomes for Rett patients using IFNγ? Would rescue/regenerative therapies with iPS-derived NPCs or viral transfer of wild-type *MECP2* gene be more efficacious? The intervention time window and a better understanding of the mode of action of IFNγ treatment and NPC transplantation therapy will be decisive to answer these questions.

In conclusion, this intriguing report builds on the work of many other groups by further unraveling *MECP2* function in the context of Rett syndrome. It suggests new possibilities for therapies and importantly, provides new research avenues that will investigate the molecular pathology of Rett syndrome in more detail.
